# The State Control of Research

**Published:** 1914-11-28

**Authors:** 


### THE STATE CONTROL OF RESEARCH.

A very important question of principle was raised when the members of the Lister Institute, at their meeting on November 18, rejected by thirty-nine votes to thirty-two the proposal of the governing body to amalgamate with the Committee for Medical Research established under the National Insurance Act and to present the Institute to the nation to form the committee's headquarters and central laboratory. A three-fourths majority was necessary in order to carry the proposal, so that the defeat is more decisive than the numbers in themselves indicate. The closeness of the voting and the weight of the names on either side show that there is much to be said for and against the proposal. The question really at issue was the advantages and dangers of the State control of research, and, keeping that fact in mind, the arguments used are worth summary.

Thus, in his opening speech in support of the governing body, Sir Henry Roscoe denied that it was intended to hand over the Institute to an official department in the sense that it was to be controlled by the political head of a department and managed by his subordinate officials. The management and control of research work was to be in the hands of a Committee of nine scientific men of approved position, with the addition of two members of the House of Commons, who would simply give an account to Parliament of how the money was spent. He regarded any attempt on the part of the House of Commons to restrict scientific investigation as unthinkable, and considered the provisions made by the Treasury as a generous offer of national help for a national need, affording an opportunity to place the Institute in a more influential and useful position than it would be likely to assume as an independent institution. The arguments in opposition were summarised in a letter published last August. The signatories—all men well known in the world of research—pointed out that the Lister is the only independent institution in England devoted to pure research in medicine, that work of the highest order has been done in it, that the valuable facilities which it afforded to independent research workers would become impossible if it had to house the nominees of a Government Committee as well as the present staff, and that experience has shown that Government controlled institutes tend to become stereotyped and inelastic. Sir E. Ray Lankester, speaking for those who were opposed to the scheme, said they were asked to amalgamate with a Committee the purpose of which was not identical with that of the Lister Institute. The Research Committee was connected with the Insurance scheme and would be concerned with its administration, while the business of the Institute was research. He would welcome a Government grant, but it was a different thing to hand over £10,000 a year to the Government.

We have summarised these arguments because, on the whole, we think the members of the Institute were right in rejecting the scheme. We will admit that it might have added to the power and influence of the Institute. In addition to the monetary assistance derived from funds provided by the National Insurance Act, about £19,000 annually, Lord Iveagh had generously offered to build a research hospital of fifty beds on lands adjacent to the Institute at Chelsea. The inducement to amalgamate was therefore a very substantial one. Nevertheless the sacrifice of independence was too great a price to pay for these advantages. We do not believe that the interference of Parliament with research work is so unthinkable as Sir Henry Roscoe considers. Our memory of Parliamentary opposition to vivisection is all against such a view. The presence of members of the House of Commons upon the governing body would bring the Institute within the arena of politics. It would be competent for any member of Parliament to question the work and policy of the Institute and the way in which the public money entrusted to it was expended. The deeply rooted distrust of officialdom in England has a very sound basis. By all means let us have official laboratories for research. We have the nucleus for them in the pathological department of the Local Government Board, and if the Medical Research Committee wishes to graft itself upon some existing institution let it amalgamate with and develop that department. But we need, in addition, an institution where the individual investigator is free to work untrammelled upon his own lines.

